# Heterogeneity in the abundance and distribution of *Ixodes ricinus* and *Borrelia burgdorferi* (*sensu lato*) in Scotland: implications for risk prediction

**DOI:** 10.1186/s13071-016-1875-9

**Published:** 2016-11-22

**Authors:** Caroline Millins, Lucy Gilbert, Paul Johnson, Marianne James, Elizabeth Kilbride, Richard Birtles, Roman Biek

**Affiliations:** 1 Institute of Biodiversity, Animal Health and Comparative Medicine, University of Glasgow, Glasgow, Scotland; 2 The Boyd Orr Centre for Population and Ecosystem Health, University of Glasgow, Glasgow, Scotland; 3 James Hutton Institute, Craigiebuckler, Aberdeen, Scotland; 4 Institute of Biological and Environmental Sciences, University of Aberdeen, Aberdeen, UK; 5 Division of Applied Medicine, University of Aberdeen, Aberdeen, UK; 6 School of Environment and Life Sciences, University of Salford, Salford, UK; 7 Present Address: Food Standards Scotland, Aberdeen, Scotland

**Keywords:** *Borrelia burgdorferi*, *Ixodes ricinus*, Risk prediction, Spatial heterogeneity, Host community

## Abstract

**Background:**

Cases of Lyme borreliosis, a vector-borne zoonosis caused by bacteria in the *Borrelia burgdorferi* (*sensu lato*) species group, have increased in recent years in Europe. Knowledge of environmental factors associated with abundance of the tick vector *Ixodes ricinus* and the pathogen *B. burgdorferi* (*s.l*.) is of interest to understand responses to environmental changes, predict variation in risk and to inform management interventions.

**Methods:**

Nineteen woodland sites across Scotland were surveyed in 2012 for *B. burgdorferi* (*s.l.*) infection in questing *I. ricinus* nymphs (*n* = 200 per site), deer abundance and vegetation. Climatic factors were extracted for each site. Six additional sites were surveyed for questing nymphs in both 2012 and 2013 (*n* = 200 per site and year) to test for variation in *B. burgdorferi* (*s.l*.) prevalence between years.

**Results:**

The mean prevalence of *B. burgdorferi* (*s.l*.) across 19 sites was 1.7% (95% CI: 1.4–2.2%; range 0–6%), all four genospecies known to be present in the UK were detected: *B. garinii*, *B. afzelii*, *B. burgdorferi* (*sensu stricto*) and *B. valaisiana*. A higher prevalence of *B. burgdorferi* (*s.l*.), higher densities of nymphs and higher densities of infected nymphs were found at sites with warmer climates, estimated with growing degree-days. No association between infection prevalence in nymphs and woodland type (semi-natural mixed *vs* coniferous) or deer density was found. At six sites sampled in 2012 and 2013, there was a significant increase in *B. afzelli* prevalence at two sites and a decrease in *B. garinii* prevalence at one site.

**Conclusions:**

This study highlights challenges for the prediction of risk of Lyme borreliosis, reflecting the sensitivity of both pathogen and vector ecology to habitat, host and climatic factors. Significant changes in the prevalence of individual genospecies at sites monitored across time are likely to be due to variability in the host community composition between years. Our results indicate the importance of monitoring dynamic variables such as reservoir host populations as well as climate and habitat factors over multiple years, to identify environmental factors associated with Lyme borreliosis risk.

**Electronic supplementary material:**

The online version of this article (doi:10.1186/s13071-016-1875-9) contains supplementary material, which is available to authorized users.

## Background

Vector-borne diseases represent a global public health burden and cause millions of cases of disease annually [1]. A number of vector-borne diseases are emerging, either from introductions into new regions or due to changing patterns of endemic disease [2]. Lyme borreliosis is an example of an emerging, endemic vector-borne disease, with an increasing geographical distribution in recent years and increased numbers of cases in many parts of Europe including Scotland [3–5]. Between 2004 and 2014, there has been a six-fold increase in the number of Lyme borreliosis cases recorded in Scotland [5]. Therefore, identifying environmental risk factors for the tick vector *Ixodes ricinus* and the bacterial pathogen *Borrelia burgdorferi* (*sensu lato*) is of importance.

Lyme borreliosis is caused by infection with spirochaete bacteria of the *B. burgdorferi* (*s.l*.) species complex. This pathogen complex is maintained amongst a large number of vertebrate hosts and ixodid ticks. There are at least 19 known genospecies of *B. burgdorferi* (*s.l.*) [6, 7]. In Scotland, four genospecies have been detected in questing ticks [8, 9]: *B. afzelii*, *B. garinii*, *B. burgdorferi* (*sensu stricto*) and *B. valaisiana*. All of these genospecies have been associated with cases of disease in humans [10, 11] although *B. afzelii* and *B. garinii* are most frequently associated with clinical disease in Europe [12]. Different genospecies of *B. burgdorferi* (*s.l*.) are associated with different clinical presentations in people [13, 14], for example *B. afzelii* is more frequently associated with dermal manifestations of disease, while *B. garinii* is more frequently detected in cases of neuroborreliosis [14]. Therefore, finer scale knowledge of the geographical distribution and abundance of each genospecies is potentially useful for public health messaging and clinical diagnosis.

Species of the *B. burgdorferi* (*s.l.*) complex infect a wide range of vertebrate reservoir hosts. Some genospecies are host generalists, while others specialise on particular taxonomic groups [15]. For example, *B. afzelli* is transmitted mainly by rodent reservoir hosts [16] and *B. garinii* and *B. valaisiana* are transmitted mainly by birds [17, 18] while *B. burgdorferi* (*s.s*.) is a generalist genospecies able to infect both birds and mammals [19, 20]. Trans-stadial infection of immature stages of ixodid ticks feeding on competent reservoir hosts is considered to be the most significant mode of transmission [21]. Co-feeding transmission between an uninfected tick feeding close to an infected tick is thought to be less significant epidemiologically [22–24]. Deer are generally considered not to transmit *B. burgdorferi* (*s.l.*) [25, 26] and deer sera has been found to be borreliacidal [27]; however some studies suggest potential for co-feeding transmission [28, 29]. Deer provide blood meals to ticks and are important in many areas as tick reproduction hosts, feeding reproductively active adult female ticks [30].

In western Europe, the main tick vector of *B. burgdorferi* (*s.l.*) is the exophilic, three-host tick *Ixodes ricinus*. Temperature, humidity and a combination of these (the saturation deficit) are the primary factors in determining conditions suitable for host-seeking behaviour [31, 32], while the community composition and abundance of hosts for blood meals are strong determinants of vector abundance and *B. burgdorferi* (*s.l.*) infection rates [33, 34]. Each of the three tick life-stages, larva, nymph and adult, takes a blood meal. In a diverse host community, different proportions of each life-stage feed on different types of host. Larvae tend to feed on all members of the host community including small mammals and birds, nymphs tend to feed mainly on medium-sized vertebrates and larger hosts, and adult ticks tend to feed on large mammal hosts such as deer and sheep (also termed ‘tick reproduction hosts’) [30].

A small number of surveys of *I. ricinus* populations and *B. burgdorferi* (*s.l.*) in the UK have indicated broad scale patterns in genospecies distribution. Bird-associated genospecies *B. garinii* and *B. valaisiana* appear to dominate in England [6, 35, 36], with some reports of rodent associated *B. afzelii* [37, 38]. In Scotland, *B. afzelii* appears to be the most common genospecies [8]. There is some evidence for spatial restriction in the distribution of *B. burgdorferi* (*s.s*.) in Scotland, as this genospecies has been found mainly in the northeast region of the country, based on one previous large-scale study of 25 sites [8]. This survey which selected sites associated with cases of Lyme borreliosis, also found a lower prevalence of *B. burgdorferi* (*s.l.*) in *I. ricinus* nymphs collected at higher altitudes [8] and a higher prevalence of infection associated with semi-natural mixed woodlands compared to coniferous forest [8, 9]. A study from northern England also found a higher prevalence of infection in *I. ricinus* nymphs sampled from semi-natural mixed woodlands compared to coniferous forests [36]. It has been suggested that the higher prevalence of infection detected in semi-natural mixed woodlands may be due to higher densities of competent reservoir hosts. In particular, there may be higher densities of small mammal hosts such as wood mice (*Apodemus sylvaticus*), bank voles (*Myodes glareolus*) and shrews (*Sorex* spp.), as well as ground-foraging birds.

The prevalence of *B. burgdorferi* (*s.l.*) and the risk of Lyme borreliosis to humans (defined as the density of infected ticks) are expected to be affected by the density of competent reservoir hosts such as rodents and birds, as well as incompetent hosts such as deer which host ticks but do not infect them with the pathogen. In comparison to data on climate and habitat, it is more challenging to collect data on the vertebrate host community, particularly during cross-sectional studies of several sites. As described above, habitat proxies have been used in previous studies to assume differences in the relative abundance of reservoir hosts (rodents and birds) between semi-natural mixed woodlands and conifer plantations. However, reservoir host populations can vary between years within habitat types, particularly rodent populations which can vary significantly in abundance between years depending on environmental conditions. Correlative studies using such proxies may have limited use if the number of infected ticks and/or the genospecies composition changes significantly over time. Therefore, studies which measure the variation in *B. burgdorferi* (*s.l.*) prevalence across time are needed.

In this study we aimed to: (i) Identify environmental variables associated with the prevalence of *B. burgdorferi* (*s.l.*) in questing *I. ricinus* nymphs and the density of *B. burgdorferi* (*s.l.*) infected nymphs at sites across Scotland. As individual genospecies may have different environmental associations (due to different reservoir hosts) we also tested for environmental associations with the most commonly detected genospecies *B. afzelii* and *B. garinii*; (ii) Compare these results to a previous study from Scotland which selected sites associated with known cases of Lyme borreliosis [8]; (iii) Test for significant variation in the prevalence of genospecies of *B. burgdorferi* (*s.l.*). between years at a subset of sites; and (iv) Identify environmental variables associated with the abundance of questing *I. ricinus* nymphs at sites across Scotland.

## Methods

### Field site selection

**Fig. 1:**
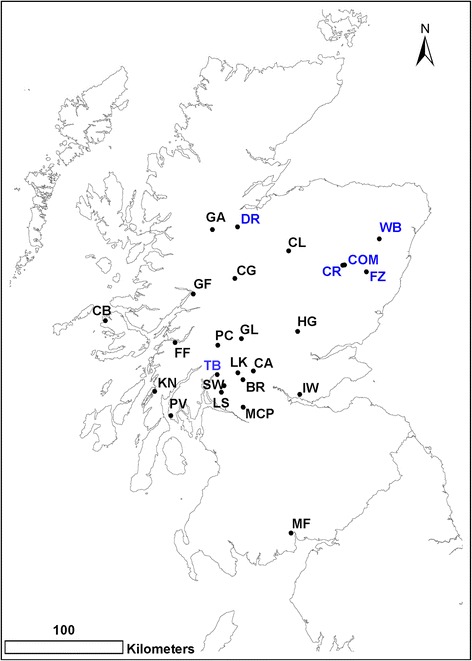
Field sites sampled for *I. ricinus* nymph abundance and *B. burgdorferi* (*s.l.*) prevalence in 2012. Sites CR, COM, WB, FZ, TB and DR shown in *blue* were sampled in multiple years (2007/2008, 2012 and 2013)

To estimate the prevalence of *B. burgdorferi* (*s.l.*) and the abundance of *I. ricinus*, 19 woodland sites were chosen across Scotland (Fig. 1, Table 1). Sites were selected to broaden the geographical area sampled from a previous survey in Scotland in 2007–2008 [8] in order to test for spatial trends in *B. burgdorferi* (*s.l.*) prevalence and genospecies composition. Sites were chosen to be from the same basic habitat types and altitudes as a previous survey in Scotland [8], and were composed of semi-natural mixed (*n* = 11) and coniferous woodland (*n* = 8). Sites were sampled between April and July in 2012.

**Table 1:** Woodland type, altitude, growing degree-days and deer dung index for 25 study sites across Scotland

Site	Year	Woodland type	Mean altitude (m)	Growing degree-days	Deer dung index 2012 (2013)
SW	2012	Coniferous	70	134,134	0.45
GL	2012	Deciduous	85	89,549	0.15
MCP	2012	Deciduous	118	125,045	0.05
LK	2012	Deciduous	300	91,168	0.05
HG	2012	Coniferous	133	130,502	0.00
BR	2012	Coniferous	89	104,415	0.10
CA	2012	Deciduous	117	122,919	0.00
CG	2012	Deciduous	262	100,233	0.00
CL	2012	Coniferous	293	105,822	0.05
FF	2012	Deciduous	47	141,376	0.00
GA	2012	Coniferous	243	95,511	0.00
GF	2012	Coniferous	130	146,246	0.00
IW	2012	Deciduous	92	135,780	0.00
KN	2012	Coniferous	75	115,821	0.10
LS	2012	Deciduous	15	142,322	0.00
PC	2012	Coniferous	250	76,429	0.05
PV	2012	Deciduous	60	122,324	0.05
CB	2012	Deciduous	30	122,197	0.20
MF	2012	Deciduous	125	na	0.00
COM	2012/3	Coniferous	154	112,953	0.00 (0.00)
CR	2012/3	Deciduous	231	96,798	0.05 (0.10)
DR	2012/3	Deciduous	82	94,397	0.00 (0.00)
TB	2012/3	Deciduous	50	127,111	0.00 (0.08)
WB	2012/3	Coniferous	200	105,889	0.00 (0.13)
FZ	2012/3	Coniferous	170	89,892	0.05 (0.00)

To investigate variability in *B. burgdorferi* (*s.l.*) prevalence and genospecies composition across time, a further six sites were selected, which were a subset of sites sampled in the previous survey [8]. These sites had been sampled between March and October one or more times in 2007 and/or 2008 and were chosen to include three sites with relatively high prevalence (mean 9.7%, range 7–14%) and three sites with relatively low prevalence (mean 1.7%, range 1–2%). For the purposes of this study, we classified these as “high prevalence” and “low prevalence” sites. These sites were re-sampled in the current study in May and June in 2012 and 2013.

### Questing tick sampling

Sampling involved slowly dragging a 1 m^2^ white blanket across the surface of the vegetation for 10 m [39]. This method samples a portion of the questing tick population, and can be used to obtain an index of questing nymph relative abundance [39]. Nymphs were recorded for statistical analysis as this life-stage is the most important for transmitting *B. burgdorferi* (*s.l.*) to humans [40]. Twenty standardized 10 m long blanket drags were carried out at each site, each drag separated by at least 50 m. Sampling was carried out between 10:00 and 16:00 h during dry conditions or at least two hours after heavy rainfall so as not to wet the blanket. Sites were sampled once per year.

### Host and environmental predictors of tick abundance and *B. burgdorferi* (*s.l*.) prevalence

Site and drag specific environmental data were recorded. Briefly, at the start of each standardized drag, the GPS location was recorded with a handheld Garmin etrex vista Hcx unit (Garmin, Schaffhausen, Switzerland) and the ground temperature and humidity measured with a RS 1360A Humidity and Temperature Meter (RS Components Ltd, Corby, UK). The mean saturation deficit (a measure of the drying power of the environment) was calculated for each site [41]. The presence or absence of deer dung was recorded in a 1 m^2^ area at the beginning and end of each 10 m blanket drag [39] and averaged by the total number of drags to create an index of deer abundance for each site [39]. The ground vegetation type and height were recorded at the start of each drag. The dominant vegetation on each drag was recorded and categorised as (i) grasses and herbaceous species; (ii) ericaceous and vaccinium species; (iii) moss species; and (iv) bracken and ferns [8]. After carrying out the 20 standardised drags at each site, additional blanket drags were added to collect a minimum of 200 nymphs per site to estimate the prevalence of *B. burgdorferi* (*s.l.*)

A number of climatic variables that could affect tick development rates or survival were extracted from GIS datasets for each site. These variables included growing degree-days (day by day sum of the number of degrees the mean temperature is greater than 4 °C, over the summer months, from long term average climate data, 1971–2000), the number of ground frost days and average annual precipitation. Climate data were derived from UK Met Office Long Term Average climate data [42]. The site locations were uploaded to ArcGIS 9.3.1 (ESRI, California, USA, 2009) and raster and polygon data files sampled at each point location using the Intersect Point tool in Hawth’s Tools [43].

### DNA extraction, *B. burgdorferi* (*s.l.*) detection and genospecies determination

The DNA from individual nymphs was extracted using an ammonia hydroxide technique [44]. *B. burgdorferi* (*s.l.*) was detected using a real time PCR as previously described [45]. Genospecies determination was by reverse line blotting [46, 47] and/or sequencing of the 5S-23S rRNA intergenic spacer region (IGS) [48]. Each trimmed IGS sequence was subjected to a basic local alignment search tool analysis (BLAST) against the National Centre for Biotechnology (NCBI) Nucleotide database. Then, each sequence was examined for polymorphisms within the IGS region which discriminate between genospecies [49].

### Statistical analysis

#### Prevalence of B. burgdorferi (s.l.) in questing nymphs and the density of infected nymphs

Using data collected from the cross sectional survey of 18 sites in 2012 (Fig. 1, Table 1; site MF was excluded as no climatic data were available), *B. burgdorferi* (*s.l.*) infection in individual nymphs (infected or uninfected) at each site was modelled using a logit link and binomial distributed errors as a function of the following site level host and environmental factors: woodland type (semi-natural mixed or coniferous), growing degree-days, nymph abundance (mean nymphs/10 m^2^) and the index of deer abundance based on deer dung, with an observation level random effect [50, 51]. An observation level random effect was incorporated to account for overdispersion; variation which is not partitioned into explanatory variables or other random effects is incorporated into this variable. As individual genospecies may have different environmental associations (due to different reservoir host associations), the described analysis was repeated individually for the two dominant genospecies *B. afzelii* and *B. garinii*.

The number of *B. burgdorferi* (*s.l.*) infected nymphs at each site was modelled with a log link and Poisson distributed errors and the following explanatory variables: woodland type (semi-natural mixed or coniferous), growing degree-days, an index of deer abundance based on dung counts and an observation level random effect. In addition, an offset variable was included in the model to account for the different area blanket dragged at each site which converted the outcome variable to the number of infected nymphs per m^2^ (density of infected nymphs). The total area blanket dragged to collect 200 nymphs was estimated by calculating the mean density of nymphs per m^2^ from the 20 standardized 10 m drags and using this to estimate the total area dragged at each site.

Data collected in the survey of six sites which were sampled in 2007/8, 2012 and 2013 were used to test whether there was a significant difference in *B. burgdorferi* (*s.l.*) prevalence between years. As the total number of nymphs tested in 2007/8 was not available, we tested for differences in *B. burgdorferi* (*s.l*.) prevalence at sites sampled in 2012 and 2013. *Borrelia burgdorferi* (*s.l.*) prevalence (infection in individual nymphs, infected or uninfected) was modelled using a logit link and binomially distributed errors with the year of sampling as an explanatory variable, and random effects of sampling site and observation. The model was re-run to test for differences in *B. afzelii* prevalence and *B. garinii* prevalence between 2012 and 2013. Other genospecies and mixed infections were present too infrequently to model separately.

The prevalence of each genospecies at individual sites and the 95% confidence interval were compared between 2012 and 2013 to see if there were significant genospecies differences at the site level between years.

#### Questing nymph abundance

Using data collected from the cross sectional survey of 18 sites in 2012 (Fig. 1, Table 1; site MF was excluded as no climatic data were available), nymph abundance (counts of nymphs per 10 m drag) was modelled using a log link and Poisson distributed errors, as a function of the following explanatory variables: ground vegetation type (at the drag level), the mean saturation deficit on the day of sampling, an index of deer abundance based on deer dung, woodland type (seminatural mixed or coniferous), growing degree-days, average annual precipitation, average number of days of ground frost (all at the site level). The model also included an interaction term between latitude and longitude of the site to test for spatial trends in questing nymph abundance. Random effects of site and at the observation level (individual drag) were included to control for inter-site variation in abundance and overdispersion, respectively [50, 51]. The ratio of residual deviance to residual degrees of freedom was calculated in the R package *RVAideMemoire* [52].

#### Model selection

All statistical analyses were carried out in R version 3.1 (R Development Core Team, Vienna, Austria) using the *lme4* package [53] for generalised linear mixed models (GLMMs). Confidence intervals were calculated using the exact binomial confidence interval in R. For all models, a maximal global model was initially fitted, which included all potential explanatory variables and specified interactions. A backward stepwise model selection was carried out based on the model’s Akaike’s Information Criterion (AIC) and variables were dropped sequentially based on their effect on the model AIC [54]. The model with the lowest AIC was selected. Initial data exploration revealed that altitude was positively correlated with growing degree-days and negatively correlated with the average number of days of ground frost. To avoid collinearity problems [55], each of these variables was included in the full model separately, in turn and the parameter associated with the best-fit model was selected for inclusion, based on the AIC as described above. For all models the AIC was corrected for small sample size (AICc) in the R package *AICcmodavg* [56].

## Results

**Fig. 2:**
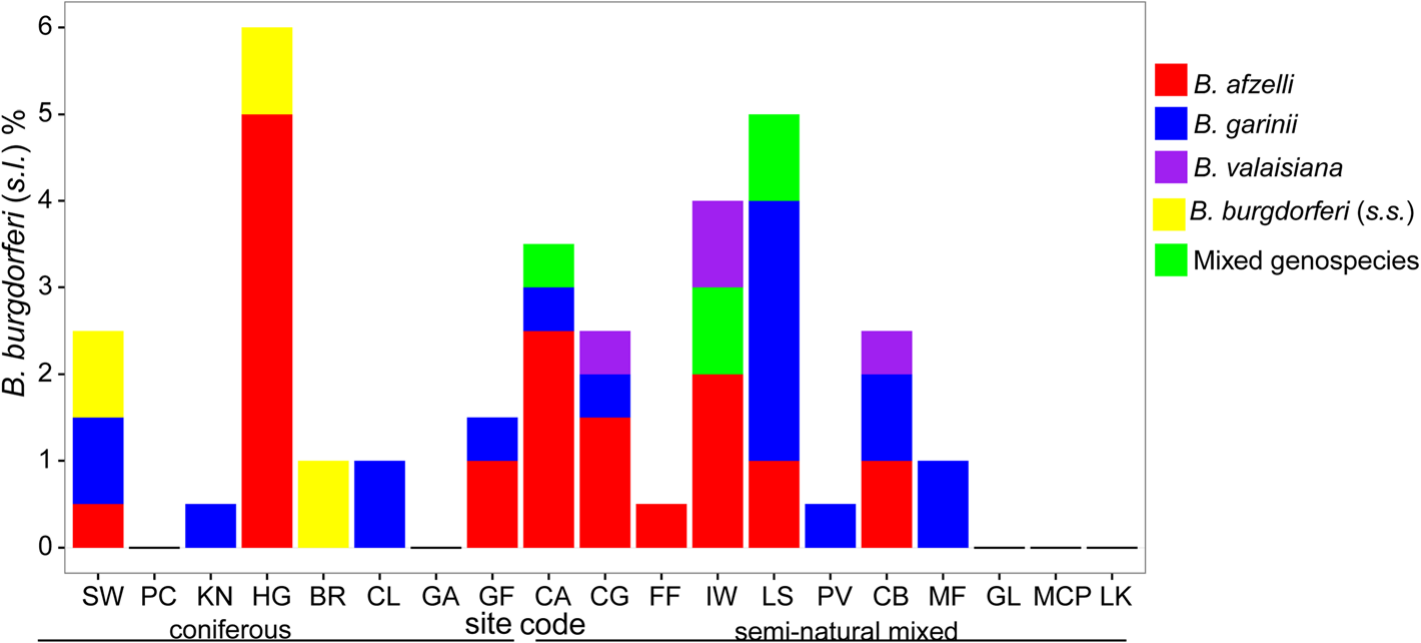
*B. burgdorferi* (*s.l.*) prevalence and genospecies composition at 19 sites in Scotland sampled in 2012 based on woodland type (coniferous or seminatural mixed). See Fig. 1 for site locations

**Fig. 3:**
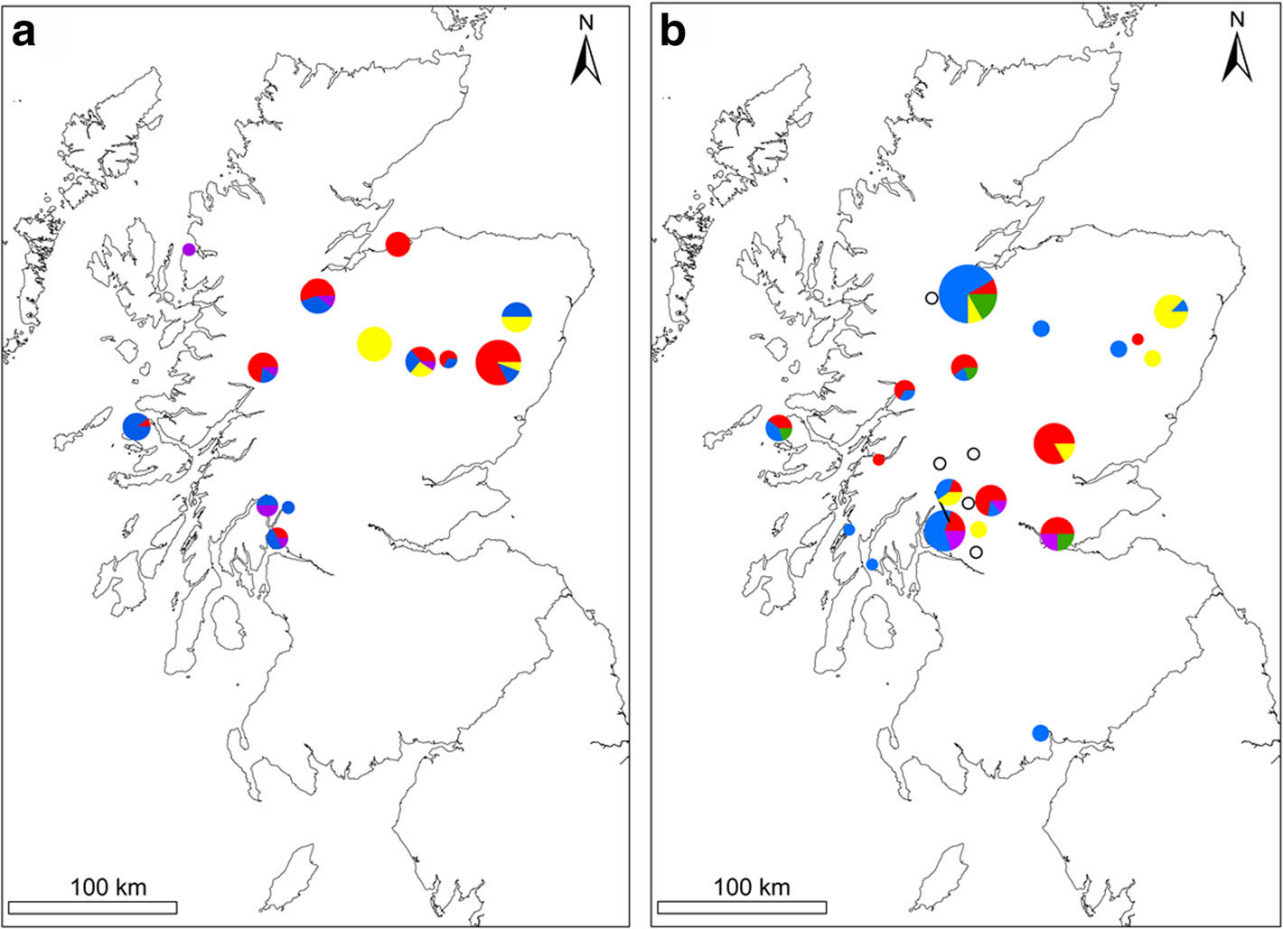
Maps to show the genospecies distribution of *B. burgdorferi* (*s.l.*) infected ticks from a previous study when ticks were sampled in 2007/2008 (**a**) [8, 9] and sites sampled in this study in 2012 (**b**). The area of the pie chart is proportional to the prevalence of *B. burgdorferi* (*s.l.*) The colour of pie chart sections corresponds to *B. burgdorferi* (*s.l.*) genospecies as follows: *red* is *B. afzelii*, *blue* is *B. garinii*, *yellow* is *B. burgdorferi* (*s.s.*), *purple* is *B. valaisiana*, *green* are mixed genospecies infections. White circles outlined in black show locations of sites where no *B. burgdorferi* (*s.l.*) infected ticks were detected

The overall prevalence of *B. burgdorferi* (*s.l.*) from the 19 sites sampled in 2012 was 1.7% (95% CI: 1.3–2.2%; range 0–6%; 66 infected ticks out of 3800 tested). Five sites had no *B. burgdorferi* (*s.l.*) infected nymphs detected (Fig. 2, Additional file 1: Table S1). Of the infected nymphs across all 19 sites 45.5% of infections were *B. afzelii*, 28.8% were *B. garinii*, 7.6% were *B. valaisiana*, 9.1% were *B. burgdorferi* (*s.s.*) and 6.1% were mixed *Borrelia* genospecies infections where two or more genospecies were detected in the same nymph. Mixed genospecies infections were composed of three *B. garinii* and *B. afzelii* infections and one combined *B. afzelii* and *B. burgdorferi* (*s.s*.) infection. The prevalence and genospecies composition of infected ticks at sites sampled in this study in 2012 and the previous study from Scotland [8] are shown in Fig. 3.

The best-fit model for *B. burgdorferi* (*s.l*.) prevalence included a positive effect of growing degree-days (delta AICc = 15.1) and a negative effect of nymph abundance (delta AICc = 7.1) (Table 2). The best-fit model for the density of infected nymphs also included a positive effect of growing degree-days (delta AICc = 11.9) as did the best-fit model for *B. afzelii* prevalence (delta AICc = 3.3). The intercept model was the best-fit model for *B. garinii* prevalence. Woodland type (seminatural mixed or coniferous) and the index of deer abundance based on deer dung were not supported as significant predictors in any of the best-fit models (Table 2).

**Table 2:** Results from the final selected generalised linear mixed model to explain variation in *B. burgdorferi* (*s.l.*) prevalence in questing nymphs, the density of *B. burgdorferi* (*s.l.*) infected nymphs and *B. afzelii* and *B. garinii* prevalence in questing nymphs

Model description	Fixed effects	Mean (estimated)	SE	*P*-value	Delta AICc
*B. burgdorferi* (*s.l.*)	(Intercept)	-9.7	1.5	9.9 × 10^-11^	–
	Growing degree-days^a^	0.5	0.1	1.8 × 10^-5^	15.1
	Mean nymph abundance/10 m^2^	-0.2	0.1	0.0010	7.1
Density of *B. burgdorferi* (*s.l.*)-infected ticks	(Intercept)	-12.4	1.8	9.6 × 10^-12^	–
*B. afzelii*	Growing degree-days^a^	0.5	0.1	0.00015	11.9
	(Intercept)	-12.1	3.3	0.00021	–
*B. garinii*	Growing degree-days^a^	0.5	0.3	0.031	3.3
	(Intercept)	-5.8	0.4	<2.0 × 10^-16^	–

Delta AICc indicates the change in AICc after removing each variable from the best-fit model

^a^UK Met Office Long Term Average climate data [35]

*Abbreviation: SE* standard error

**Fig. 4:**
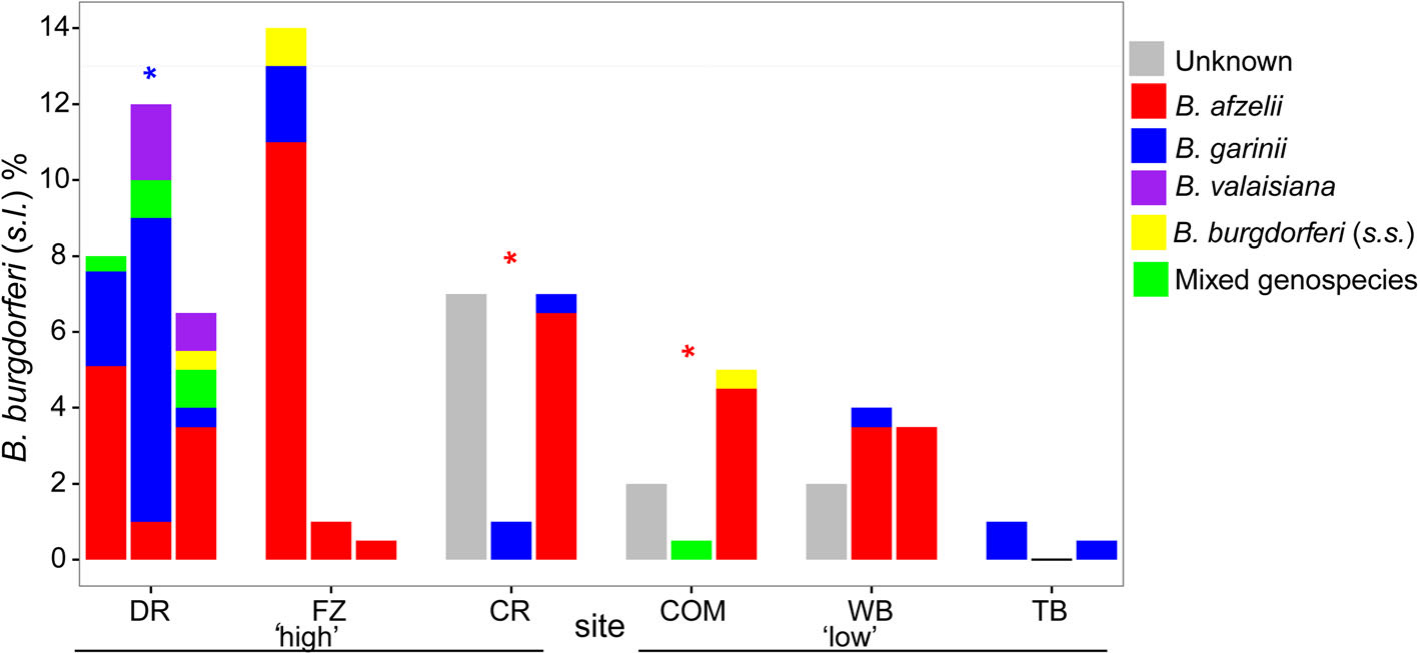
*B. burgdorferi* (*s.l.*) prevalence and genospecies composition in questing *Ixodes ricinus* nymphs at six sites in Scotland sampled in multiple years. Site locations are shown in Fig. 1. Each site has three bars which illustrate the results of sampling conducted in (from left to right for each site) 2007/2008, 2012 and 2013. Sites are categorised as ‘high’ and ‘low’ prevalence based on the initial prevalence estimate in 2007/2008

At the six sites sampled in multiple years, (1200 nymphs tested in each year), the prevalence at ‘low’ and ‘high’ prevalence sites varied through time (Fig. 4, Additional file 2: Table S2). The best-fit model for *B. burgdorferi* (*s.l*.), *B. afzelii* and *B. garinii* prevalence at the six sites sampled in 2012 and 2013 was the intercept model, year of sampling was not supported in any of these models (Additional file 3: Table S3). There was a non-significant tendency for an increased prevalence of *B. afzelii* in 2013 compared to 2012 (delta AICc = 1.6, *P* = 0.06, Additional file 3: Table S3). At the site level, there were significant increases in *B. afzelii* prevalence at two of the six sites, ‘COM’ and ‘CR’ and a significant decrease in the prevalence of *B. garinii* at site DR between 2012 and 2013 (Fig. 4).

The mean number of nymphs per 10 m^2^ blanket drag at each site ranged between 0.6–11.5 nymphs (Additional file 1: Table S1). The best-fit model for nymph abundance included growing degree-days, and the ground vegetation type. Inclusion of an observation level random effect improved the model fit and absorbed overdispersion in the data [50, 51]. The ratio of the residual deviance to the residual degrees of freedom was 0.6 with the observation level random effect, compared to 3.2 without it. Increased numbers of nymphs were found with higher growing degree-days (delta AICc = 2.5) and in areas where the forest floor was dominated by grasses compared to all other ground vegetation types (delta AICc = 2.7) (Additional file 4: Table S4). The index of deer abundance based on deer dung, the saturation deficit, woodland type (seminatural mixed or coniferous) and an interaction term between latitude and longitude of the site were not supported as significant predictors in the best-fit model.

## Discussion

The overall genospecies composition in this study was consistent with previous data from Scotland [9]. *Borrelia afzelii* was the most common genospecies detected in questing nymphs, followed by *B. garinii* and both *B. valaisiana* and *B. burgdorferi* (*s.s*.) were found at lower prevalences. Consistent with previous reports [9, 36], *B. valaisiana* was detected only in seminatural mixed woodland and *B. burgdorferi* (*s.s*.) was detected only in coniferous woodland. Our study also showed a wider distribution for *B. burgdorferi* (*s.s*.) than previously described, with detection of this genospecies in central and western Scotland as well as the northeast Highlands (Fig. 3). *Borrelia valaisiana* is associated with bird hosts, and there are many potential competent reservoir hosts among bird communities in semi-natural mixed woodlands [57]. *Borrelia burgdorferi* (*s.s*.) is a generalist genospecies, able to infect both birds and mammals. Several studies have suggested an association between red squirrels (a conifer specialist) and *B. burgdorferi* (*s.s*.) [9, 58, 59] but their contribution in proportion to other hosts in maintaining this genospecies remains unknown. The significance of red squirrels as a reservoir host for *B. burgdorferi* (*s.s*.) warrants further investigation as this could be useful in predicting the distribution of this genospecies.

The overall prevalence of *B. burgdorferi* (*s.l.*) in this study (mean: 1.7%; 95% CI: 1.3–2.2%; range: 0–6%) is significantly lower than previously reported in a study of 25 woodland sites around Scotland (mean: 5.6%; 95% CI: 4.4–7.0%; range: 0.8–13.9%) [8], and within the lower quartile of the range of prevalence reported across Europe [60]. The previous survey of ticks and *B. burgdorferi* (*s.l.*) in Scotland selected woodland sites thought to be associated with cases of Lyme borreliosis from questionnaire surveys [8, 61]. The difference in prevalence between these studies may be due to the method of site section, the geographic area which was sampled, differences in PCR testing methodology or reflect real differences in *B. burgdorferi* (*s.l.*) circulation between years (e.g. due to fluctuations in reservoir host populations). A further difference from the previous study [8] was that we did not detect *B. burgdorferi* (*s.l.*) at five of our study sites, based on a sample size of 200 nymphs per site. This suggests absence of *B. burgdorferi* (*s.l.*) or persistence at very low levels (mean: 0%, 95% CI: 0–1.8%). This is an unusual finding; in a meta-analysis of European studies, only two out of 110 studies found a prevalence less than 1.8% [62]. Another study from the UK carried out in northern England also did not detect *B. burgdorferi* (*s.l.*) in ticks tested from four of 11 sites [36].

A number of possible mechanisms could contribute to a lower prevalence and an apparent absence of *B. burgdorferi* (*s.l.*) transmission at some sites in this study. These include a high density of incompetent hosts such as deer, low densities of reservoir hosts such as rodents and birds, and host vector interactions such as lower numbers of nymphs on reservoir hosts [15]. We did not find a relationship between the index of deer abundance and *B. burgdorferi* (*s.l.*) prevalence, unlike a previous survey from Scotland [8] which reported a positive relationship between red deer dung and *B. burgdorferi* (*s.l.*) prevalence. The relationship between the density of deer and *B. burgdorferi* (*s.l.*) prevalence can be variable based on theoretical [15, 63] and empirical studies, with positive [8], negative [64, 65] and no effect of deer density on *B. burgdorferi* (*s.l.*) prevalence [66, 67] reported in field studies. Lower *B. burgdorferi* (*s.l.*) prevalence was associated with higher nymph abundance in this study. This may indirectly suggest a role for high densities of incompetent hosts such as deer which act as tick reproduction hosts and can ‘dilute’ the prevalence of *B. burgdorferi* (*s.l.*) if high numbers of immature ticks feed on them [15, 30, 63]. However, unlike previous studies [8, 39, 68, 69], an index of deer density based on deer dung was not supported in the nymph abundance model in this study. Although using a method to estimate deer abundance which had been used successfully in previous studies from the same area [8, 39], we suspect that index of relative deer abundance in this study is not well correlated with actual deer abundance. We counted low amounts of deer dung or an absence of dung at sites which other evidence of deer presence (e.g. tracks) were seen. To improve measures of deer abundance in future studies, it may be useful to examine a larger area at each site for deer dung.

Semi-natural mixed woodland has been associated with an increased prevalence of *B. burgdorferi* (*s.l.*) in nymphs compared to coniferous plantation in previous studies in the UK [8, 9, 36]; however we did not find any significant association between the prevalence of *B. burgdorferi* (*s.l.*) and woodland type. It is possible that the low prevalence in this study may have decreased our power to detect an effect of woodland type. A consequence of reduced power is decreased confidence in interpreting explanatory variables which are not maintained in the model due to an increased chance of type ll errors. In contrast, when significant effects are detected, such as the association between growing degree-days and prevalence in this study, this shows strong support for these explanatory variables (Table 2). In this study, a higher prevalence of *B. burgdorferi* (*s.l.*), *B. afzelii* and a higher density of infected nymphs were found at sites with warmer climates, as these may be the areas which are more suitable for rodent reservoir hosts. A higher likelihood of finding infected ticks was reported at lower altitudes in previous studies in Scotland [8] and Switzerland [70]. Temperature is the main abiotic factor which varies with altitude [39], with warmer climates present at lower altitudes. So, these studies also suggest that climatic factors (either directly, or as a proxy of rodent host abundance) affect the prevalence of *B. burgdorferi* (*s.l.*) in questing ticks.

Whether local prevalence and the density of infected ticks remains stable over time is an important consideration for developing models to predict variation in risk-based on long-term averaged abiotic and habitat factors. By sampling woodland sites in multiple years, we found that the *B. burgdorferi* (*s.l.*) prevalence and genospecies composition at individual sites initially categorised as ‘high’ and ‘low’ prevalence can be highly variable between years (Fig. 4). Sampling variation could contribute to a proportion of the observed variation, however the sample size of nymphs collected at each site (*n* = 200) was chosen to be high to minimize this. Variation in prevalence between years has also been reported at sites sampled over several years in Europe [71, 72] and North America [73], although a recent study in the Netherlands found surprisingly little inter-year variation at all sites [74].

Between-year variation in the prevalence and genospecies composition of *B. burgdorferi* (*s.l.*) in questing ticks is most likely to be due to changes in the vertebrate host community composition. For example, a study from North America has shown that the densities of mice and chipmunks were found to be a significant predictor of the numbers of infected ticks the following year [73]. This suggests that in order to develop methods to predict areas of ‘high’ and ‘low’ environmental risk for humans, measures of both dynamic environmental factors such as host population densities will need to be measured as well as more static factors such as long term averaged climatic factors, habitat and vegetation. As *I. ricinus* life-stages generally take one blood meal per year, then emerge after developing into the next stage the following year, we would expect the prevalence of infection in nymphs to be affected by reservoir host densities in the previous year.

For the six sites sampled repeatedly in subsequent years, we found a non-significant trend towards higher numbers of nymphs infected with *B. afzelii* in 2013 compared to 2012 (Additional file 3: Table S3). At the individual site level, there was a significant increase in *B. afzelii* at two sites in 2013 compared to 2012 (Fig. 4). Another longitudinal study carried out in the Netherlands, in which *B. afzelii* was the dominant genospecies, found an alternating annual trend in prevalence at three out of four sites [71]. A decrease in *B. afzelii* prevalence between consecutive years was also noted at several sites in a study from Germany [72]. Rodent species are the most competent hosts for *B. afzelii* [16]. Parallel fluctuations in the prevalence of *B. afzelii* at several sites could be due to changes in rodent populations, perhaps in response to similar environmental conditions between these sites. None of these studies including the current study, monitored rodent abundance along with the prevalence of infection in questing ticks. Such studies are needed to test whether variability in small mammal density affects prevalence in questing ticks as has been described in North America [73].

Sites with a higher growing degree-day index generally had higher densities of questing nymphs, most likely due to effects on temperature-dependent inter-stadial development rates, survival rates, oviposition and egg development rates and levels of activity [75, 76]. Unlike other studies which have reported a positive effect of deer density on tick abundance [8, 68], an index of deer abundance (from dung counts) was not supported in models of tick abundance in this study. We suspect that the index of deer abundance used in this study may be poorly correlated with actual deer abundance as discussed above. The saturation deficit on the day of sampling was also not supported in this study in the best-fit model of tick abundance. A mean value for the saturation deficit over the previous 30 days prior to tick sampling may be a more useful predictor than sampling this parameter on one day [41]. We found that ground vegetation dominated by grass was associated with the highest density of questing nymphs compared to all other ground vegetation types. The category of ground vegetation associated with the highest tick densities varies between studies and geographic areas in Scotland using the same methodology. A previous study showed that bracken had lower tick counts on blanket drags compared to all other vegetation types [8]. A focal study around Loch Lomond in Scotland, found higher tick counts on blanket drags in ericaceous vegetation [77]. Thus, ground vegetation under woodland canopies appears to have limited predictive power for tick abundance over larger geographic areas.

## Conclusions

We found that a higher prevalence of *B. burgdorferi* (*s.l.*), a higher prevalence of *B. afzelii*, higher nymph abundance and higher densities of ticks infected with *B. burgdorferi* (*s.l.*) (a measure of Lyme borreliosis risk) were associated with warmer sites in Scotland. In contrast to a previous study in Scotland [8], we did not find an association between *B. burgdorferi* (*s.l.*) prevalence and woodland type. We also did not find a relationship between an index of deer abundance (based on deer dung counts) and the abundance of *I. ricinus*, *B. burgdorferi* (*s.l.*) prevalence or the density of infected nymphs, as previously reported [8, 68]. However, we suspect that the index of deer abundance used in this study may not be well correlated with actual deer abundance and examining an increased area for the presence of deer dung may be beneficial in future studies. The prevalence of *B. afzelii* and *B. garinii* varied significantly at three of the six sites monitored over time, but no consistent trend in prevalence was seen across all sites. This suggests that there are changes in host communities and the proportion of competent reservoir hosts at sites over time, and that changes in host densities varies between locations. This emphasises the difficulty in forming generic rules of environmental associations with this suite of pathogens. Future challenges to predicting patterns of spatial and temporal variability in Lyme borreliosis risk depend on being able to directly or indirectly incorporate measures of the host community as well as other more static biotic and abiotic factors.

## Additional files

Additional file 1: Table S1. Nymph abundance, and *B. burgdorferi* (*s.l.*) prevalence and individual genospecies prevalence at 19 sites visited between April and July 2012 (*n* = 200 nymphs at each site). (DOCX 17 kb)
Additional file 2: Table S2.
*B. burgdorferi* (*s.l.*) prevalence and genospecies prevalence at each site sampled in, 2007/2008, 2012 and 2013. A full set of genospecies data and sample size to calculate the 95% CI were not available for all sites sampled in 2007/2008. (DOCX 17 kb)
Additional file 3: Table S3. Results for the final generalised linear mixed model to explain variation in the prevalence of infected nymphs collected at six sites sampled in 2012 and 2013. (DOCX 14 kb)
Additional file 4: Table S4. Results from the final selected generalised linear mixed model to explain variation in questing *Ixodes ricinus* nymph abundance. Delta AICc indicates the change in AICc after removing each variable from the best-fit model. (DOCX 14 kb)

## Abbreviations

AIC: Akaike’s Information Criterion
BLAST: Basic local alignment search tool analysis
IGS: Intergenic spacer region
NCBI: National Centre for Biotechnology

## References

[1] Gubler DJ (2009). Vector-borne diseases. Rev Sci Tech.

[2] Kilpatrick AM, Randolph SE (2012). Drivers, dynamics, and control of emerging vector-borne zoonotic diseases. Lancet.

[3] Linard C, Lamarque P, Heyman P, Ducoffre G, Luyasu V, Tersago K (2007). Determinants of the geographic distribution of Puumala virus and Lyme borreliosis infections in Belgium. Int J Health Geogr.

[4] Quine CP, Barnett J, Dobson ADM, Marcu A, Marzano M, Moseley D (2011). Frameworks for risk communication and disease management: the case of Lyme disease and countryside users. Philos Trans R Soc Lond B Biol Sci.

[5] Health Protection Scotland. Lyme Disease, Scotland, Annual Totals. http://www.hps.scot.nhs.uk/resourcedocument.aspx?resourceid=1455. Accessed 26 Apr 2015.

[6] Margos G, Vollmer SA, Ogden NH, Fish D (2011). Population genetics, taxonomy, phylogeny and evolution of *Borrelia burgdorferi* sensu lato. Infect Genet Evol.

[7] Ivanova LB, Tomova A, González-Acuña D, Murúa R, Moreno CX, Hernández C (2014). *Borrelia chilensis*, a new member of the *Borrelia burgdorferi* sensu lato complex that extends the range of this genospecies in the Southern Hemisphere. Environ Microbiol.

[8] James MC, Bowman AS, Forbes KJ, Lewis F, McLeod JE, Gilbert L (2013). Environmental determinants of *Ixodes ricinus* ticks and the incidence of *Borrelia burgdorferi* sensu lato, the agent of Lyme borreliosis, in Scotland. Parasitology.

[9] James MC, Gilbert L, Bowman AS, Forbes KJ (2014). The heterogeneity, distribution, and environmental associations of *Borrelia burgdorferi* sensu lato, the agent of Lyme borreliosis, in Scotland. Front Public Health.

[10] Rijpkema SGT, Tazelear DJ, Molkeboer HJ, Noordhoek GT, Plantinga G, Schouls LM (1997). Detection of *Borrelia afzelii*, *Borrelia burgdorferi* sensu stricto, *Borrelia garinii* and group VS116 by PCR in skin biopsies of patients with erythema migrans and acrodermatitis chronica atrophicans. Clin Microbiol Infect.

[11] Diza E, Papa A, Vezyri E, Tsounis S, Milonas I, Antoniadis A (2004). *Borrelia valaisiana* in cerebrospinal fluid. Emerg Infect Dis.

[12] Stanek G, Reiter M (2011). The expanding Lyme *Borrelia* complex-clinical significance of genomic species?. Clin Microbiol Infect.

[13] van Dam AP, Kuiper H, Vos K, Widjojokusumo A, de Jongh BM, Spanjaard L (1993). Different genospecies of *Borrelia burgdorferi* are associated with distinct clinical manifestations of Lyme borreliosis. Clin Infect Dis.

[14] Strle F, Stanek G (2009). Clinical manifestations and diagnosis of Lyme borreliosis. Curr Probl Dermatol.

[15] Mannelli A, Bertolotti L, Gern L, Gray J (2012). Ecology of *Borrelia burgdorferi sensu lato* in Europe: transmission dynamics in multi-host systems, influence of molecular processes and effects of climate change. FEMS Microbiol Rev.

[16] Hanincová K, Schäfer SM, Etti S, Sewell HS, Taragelová V, Ziak D (2003). Association of *Borrelia afzelii* with rodents in Europe. Parasitology.

[17] Hanincová K, Taragelová V, Koci J, Schäfer SM, Hails R, Ullmann AJ (2003). Association of *Borrelia garinii* and *B. valaisiana* with Songbirds in Slovakia. Appl Environ Microbiol.

[18] Taragel’ová V, Koči J, Hanincová K, Kurtenbach K, Derdáková M, Ogden NH (2008). Blackbirds and song thrushes constitute a key reservoir of *Borrelia garinii*, the causative agent of borreliosis in central Europe. Appl Environ Microbiol.

[19] Baum E, Hue F, Barbour AG (2012). Experimental infections of the reservoir species *Peromyscus leucopus* with diverse strains of *Borrelia burgdorferi*, a Lyme disease agent. MBio.

[20] Richter D, Spielman A, Komar N, Matuschka F-R (2000). Competence of American robins as reservoir hosts for Lyme disease spirochetes. Emerg Infect Dis.

[21] Gern L, Humair P-F, Gray J, Kahl O, Lane R, Stanek G (2002). Lyme Borreliosis, Biol. Epidemiol. Control.

[22] Ogden NH, Nuttall PA, Randolph SE (1997). Natural Lyme disease cycles maintained via sheep by co-feeding ticks. Parasitology.

[23] Gern L, Rais O (1996). Efficient transmission of *Borrelia burgdorferi* between cofeeding *Ixodes ricinus* ticks (Acari: Ixodidae). J Med Entomol.

[24] Voordouw MJ (2015). Co-feeding transmission in Lyme disease pathogens. Parasitology.

[25] Jaenson TGT, Talleklint L (1992). Incompetance of roe deer as reservoirs of the Lyme borreliosis spirochaete. J Med Entomol.

[26] Telford SR, Mather TN, Moore SI, Wilson ML, Spielman A (1988). Incompetence of deer as reservoirs of the Lyme disease spirochete. Am J Trop Med Hyg.

[27] Kurtenbach K, Sewell H-S, Ogden NH, Randolph SE, Nuttall PA (1998). Serum Complement sensitivity as a key factor in Lyme disease ecology. Infect Immun.

[28] Kimura K, Isogai E, Isogai H, Kamewaka Y, Nishikawa T, Ishii N (1995). Detection of Lyme disease spirochaetes in the skin of naturally infected wild sika deer (*Cervus nippon yesoensis*). Appl Environ Microbiol.

[29] Oliver JH, Stallknecht D, Chandler FH, James AM, McGuire BS, Howerth E (1992). Detection of *Borrelia burgdorferi* in laboratory-reared *Ixodes dammini* (Acari: Ixodidae) fed on experimentally inoculated white-tailed deer. J Med Entomol.

[30] Gray JS (1998). The ecology of ticks transmitting Lyme borreliosis. Exp Appl Acarol.

[31] Randolph S, Green RM, Hoodless A, Peacey MF (2002). An empirical quantitative framework for the seasonal population dynamics of the tick *Ixodes ricinus*. Int J Parasitol.

[32] Tagliapietra V, Rosà R, Arnoldi D, Cagnacci F, Capelli G, Montarsi F (2011). Saturation deficit and deer density affect questing activity and local abundance of *Ixodes ricinus* (Acari, Ixodidae) in Italy. Vet Parasitol.

[33] LoGiudice K, Ostfeld RS, Schmidt KA, Keesing F (2003). The ecology of infectious disease: Effects of host diversity and community composition on Lyme disease risk. Proc Natl Acad Sci U S A.

[34] LoGiudice K, Duerr STK, Newhouse MJ, Schmidt KA, Killilea ME, Ostfeld RS (2008). Impact of host community composition on Lyme disease risk. Ecology.

[35] Kurtenbach K, Peacey M, Rijpkema SGT, Hoodless AN, Nuttall PA, Randolph SE (1998). Differential transmission of the genospecies of *Borrelia burgdorferi* sensu lato by game birds and small rodents in England. Appl Environ Microbiol.

[36] Bettridge J, Renard M, Zhao F, Bown KJ, Birtles RJ (2013). Distribution of *Borrelia burgdorferi* sensu lato in *Ixodes ricinus* populations across central Britain. Vector-Borne Zoonotic Dis.

[37] Vollmer SA, Bormane A, Dinnis RE, Seelig F, Dobson ADM, Aanensen DM (2011). Host migration impacts on the phylogeography of Lyme borreliosis spirochaete species in Europe. Environ Microbiol.

[38] Hansford KM, Fonville M, Jahfari S, Sprong H, Medlock JM (2014). *Borrelia miyamotoi* in host-seeking *Ixodes ricinus* ticks in England. Epidemiol Infect.

[39] Gilbert L (2010). Altitudinal patterns of tick and host abundance: a potential role for climate change in regulating tick-borne diseases?. Oecologia (Berlin).

[40] Robertson JN, Gray JS, Stewart P (2000). Tick bite and Lyme borreliosis risk at a recreational site in England. Eur J Epidemiol.

[41] Perret JL, Guigoz E, Rais O, Gern L (2000). Influence of saturation deficit and temperature on *Ixodes ricinus* tick questing activity in a Lyme borreliosis-endemic area (Switzerland). Parasitol Res.

[42] Met Office. Long Term Average Climate Data 2009. http://www.metoffice.gov.uk/climatechange/science/monitoring/ukcp09/download/longterm/fivekm_monthly.html. Accessed 30 Apr 2015.

[43] Beyer HL. Hawth’s Tools. 2004. http://www.spatialecology.com/htools/. Accessed 30 Apr 2015.

[44] Gern L, Douet V, López Z, Rais O, Cadenas FM (2010). Diversity of *Borrelia* genospecies in *Ixodes ricinus* ticks in a Lyme borreliosis endemic area in Switzerland identified by using new probes for reverse line blotting. Ticks Tick Borne Dis.

[45] Courtney JW, Kostelnik LM, Zeidner NS, Massung RF (2004). Multiplex Real-Time PCR for Detection of *Anaplasma phagocytophilum* and *Borrelia burgdorferi*. J Clin Microbiol.

[46] Alekseev AN, Dubinina HV, Van De PI, Schouls LM (2001). Identification of *Ehrlichia* spp.and *Borrelia burgdorferi* in *Ixodes* ticks in the Baltic regions of Russia. J Clin Microbiol.

[47] Poupon M, Lommano E, Douet V, Rais O, Schaad M, Jenni L (2006). Prevalence of *Borrelia burgdorferi* sensu lato in ticks collected from migratory birds in Switzerland. Appl Environ Microbiol.

[48] Rijpkema SG, Molkenboer MJ, Schouls LM, Jongejan F, Schellekens J (1995). Simultaneous detection and genotyping of three genomic groups of *Borrelia burgdorferi* sensu lato in Dutch *Ixodes ricinus* ticks by characterization of the amplified intergenic spacer region between 5S and 23S rRNA genes. J Clin Microbiol.

[49] Millins C, Magierecka A, Gilbert L, Edoff A, Brereton A, Kilbride E (2015). An invasive mammal (gray squirrel, *Sciurus carolinensis*) commonly hosts diverse and atypical genotypes of the zoonotic pathogen *Borrelia burgdorferi* sensu lato. Appl Environ Microbiol.

[50] Harrison XA (2014). Using observation-level random effects to model overdispersion in count data in ecology and evolution. PeerJ.

[51] Elston DA, Moss R, Boulinier T, Arrowsmith C, Lambin X (2001). Analysis of aggregation, a worked example: numbers of ticks on red grouse chicks. Parasitology.

[52] Hervé M. ‘RVAideMemoire’. 2016. https://cran.r-project.org/web/packages/RVAideMemoire/index.html. Accessed 1 July 2016.

[53] Bates D, Maechler M, Bolker B, Walker S. lme4: Linear mixed-effects, models using Eigen and S4. 2014. http://cran.r-project.org/package=lme4. Accessed 12 Apr 2015.

[54] Chambers JM. Chapter 4. Linear Models. In: Chambers JM, Hastie TJ, editors. Stat. Model. S. Pacific Grove: Wadsworth & Brooks/Cole; 1992. p. 100–45.

[55] Estrada-Peña A, Gray JS, Kahl O, Lane RS, Nijhof AM. Research on the ecology of ticks and tick-borne pathogens-methodological principles and caveats. Front. Cell. Infect. Microbiol. 2013;3:. doi:10.3389/fcimb.2013.00029. 10.3389/fcimb.2013.00029 PMC3737478 23964348

[56] Mazerolle MJ. Model Selection and Multimodel Inference Based on (Q) AIC (c). 2015. https://cran.r-project.org/web/packages/AICcmodavg/AICcmodavg.pdf. Accessed 15 Apr 2015.

[57] James MC, Furness RW, Bowman AS, Forbes KJ, Gilbert L (2011). The importance of passerine birds as tick hosts and in the transmission of *Borrelia burgdorferi*, the agent of Lyme disease: a case study from Scotland. Ibis.

[58] Humair P-F, Gern L (1998). Relationship between *Borrelia burgdorferi* sensu lato species, red squirrels (*Sciurus vulgaris*) and *Ixodes ricinus* in enzootic areas in Switzerland. Acta Trop.

[59] Pisanu B, Chapuis J-L, Dozières A, Basset F, Poux V, Vourc’h G (2014). High prevalence of *Borrelia burgdorferi* s.l. in the European red squirrel *Sciurus vulgaris* in France. Ticks Tick Borne Dis.

[60] Estrada-Peña A, Ortega C, Sánchez N, DeSimone L, Sudre B, Suk JE (2011). Correlation of *Borrelia burgdorferi* sensu lato prevalence in Questing *Ixodes ricinus* ticks with specific abiotic traits in the Western Palearctic. Appl Environ Microbiol.

[61] James MC (2010). The ecology, genetic diversity and epidemiology of Lyme borreliosis in Scotland. PhD thesis.

[62] Rauter C, Hartung T (2005). Prevalence of *Borrelia burgdorferi* sensu lato genospecies in *Ixodes ricinus* ticks in Europe: a metaanalysis. Appl Environ Microbiol.

[63] Rosà R, Pugliese A, Norman R, Hudson PJ (2003). Thresholds for disease persistence in models for tick-borne infections including non-viraemic transmission, extended feeding and tick aggregation. J Theor Biol.

[64] Mysterud A, Easterday WR, Qviller L, Viljugrein H, Ytrehus B (2013). Spatial and seasonal variation in the prevalence of *Anaplasma phagocytophilum* and *Borrelia burgdorferi* sensu lato in questing *Ixodes ricinus* ticks in Norway. Parasit Vectors.

[65] Gray JS, Kahl O, Janetzki C, Stein J (1992). Studies on the ecology of Lyme disease in a deer forest in County Galway. Ireland J Med Entomol.

[66] Pichon B, Mousson L, Figureau C, Rodhain F, Perez-Eid C (1999). Density of deer in relation to the prevalence of *Borrelia burgdorferi* s.l. in *Ixodes ricinus* nymphs in Rambouillet forest, France. Exp Appl Acarol.

[67] Werden L, Barker IK, Bowman J, Gonzales EK, Leighton PA, Lindsay LR (2014). Geography, deer, and host biodiversity shape the pattern of Lyme disease emergence in the Thousand Islands Archipelago of Ontario, Canada. PLoS ONE.

[68] Gilbert L, Maffey GL, Ramsay SL, Hester A (2012). The effect of deer management on the abundance of *Ixodes ricinus* in Scotland. Ecol Appl.

[69] Ruiz-Fons F, Gilbert L (2010). The role of deer as vehicles to move ticks, *Ixodes ricinus*, between contrasting habitats. Int J Parasitol.

[70] Morán Cadenas F, Rais O, Jouda F, Douet V, Humair P, Moret J (2007). Phenology of *Ixodes ricinus* and infection with *Borrelia burgdorferi* sensu lato along a North- and South-facing altitudinal gradient on Chaumont Mountain. Switzerland J Med Entomol.

[71] Wielinga PR, Gaasenbeek C, Fonville M, De Boer A, De Vries A, Dimmers W (2006). Longitudinal analysis of tick densities and *Borrelia*, *Anaplasma*, and *Ehrlichia* infections of *Ixodes ricinus* ticks in different habitat areas in the Netherlands. Appl Environ Microbiol.

[72] Schwarz A, Hönig V, Vavrušková Z, Grubhoffer L, Balczun C, Albring A (2012). Abundance of *Ixodes ricinus* and prevalence of *Borrelia burgdorferi* s.l. in the nature reserve Siebengebirge, Germany, in comparison to three former studies from 1978 onwards. Parasit Vectors.

[73] Ostfeld RS, Canham CD, Oggenfuss K, Winchcombe RJ, Keesing F (2006). Climate, deer, rodents, and acorns as determinants of variation in Lyme-disease risk. PLoS Biol.

[74] Takken W, van Vliet AJH, Verhulst NO, Jacobs F, Gassner F, Hartemink N, et al. Acarological risk of *Borrelia burgdorferi* sensu lato infections across space and time in The Netherlands. Vector-Borne and Zoon Dis. 2016. (in press). 10.1089/vbz.2015.1933 27893309

[75] Macleod J (1935). *Ixodes ricinus* in relation to its physical environment III. Climate and reproduction. Parasitology.

[76] Dobson ADM, Randolph SE (2011). Modelling the effects of recent changes in climate, host density and acaricide treatments on population dynamics of *Ixodes ricinus* in the UK. J Appl Ecol.

[77] Millins CL (2016). Ecological drivers of a vector borne pathogen: Distribution and abundance of *Borrelia burgdorferi* sensu lato and its vector *Ixodes ricinus* in Scotland. PhD thesis.

