# Supplementary material for: Heterogeneity in the abundance and distribution of Ixodes ricinus and Borrelia burgdorferi (sensu lato) in Scotland: implications for risk prediction
Source: Parasit Vectors. 2016 Nov 22;9:595. doi: 10.1186/s13071-016-1875-9 (PMC5120507; doi:10.1186/s13071-016-1875-9)
Supplement: Additional file 1: Table S1. — Nymph abundance, and B. burgdorferi (s.l.) prevalence and individual genospecies prevalence at 19 sites visited between April and July 2012 (n = 200 nymphs at each site). (DOCX 17 kb) [file 13071_2016_1875_MOESM1_ESM.docx]

**Additional file 1. Table S1.** Nymph abundance, and *Borrelia burgdorferi* (*sensu lato*) prevalence and individual genospecies prevalence at 19 sites visited between April and July 2012 (*n* = 200 nymphs at each site). *Abbreviations*: Bb (*s.l*.) % = overall *B. burgdorferi* (*s.l*.) prevalence, B. val % = *B. valaisiana*, B. gar % = *B. garinii*, B. afz % = *B. afzelii*, B (s.s.) = *B. burgdorferi* (*sensu stricto*), Mixed % = mixed *B. burgdorferi* (*s.l*.) genospecies

| **Site** | **Mean nymphs/10 m^2^ (SD)** | **Bb (*s.l*.) % (95% CI)** | **B. val % (*n*)** | **B. gar % (*n*)** | **B. afz % (*n*)** | **B (*s.s*.) % (*n*)** | **Mixed % (*n*)** |
| --- | --- | --- | --- | --- | --- | --- | --- |
| SW | 5.7 (4.7) | 2.5 (0.8–5.7) | 0 | 1.0 (2) | 0.5 (1) | 1 (2) | 0 |
| GL | 2.6 (2.4) | 0.0 (0.0–1.8) | 0 | 0 | 0 | 0 | 0 |
| MCP | 10.6 (22.4) | 0.0 (0.0–1.8) | 0 | 0 | 0 | 0 | 0 |
| LK | 2.1 (2.3) | 0.0 (0.0–1.8) | 0 | 0 | 0 | 0 | 0 |
| HG | 1.8 (2.2) | 6.0 (3.1–10.2) | 0 | 0 | 5 (10) | 1 (2) | 0 |
| BR | 1.5 (2.2) | 1.0 (0.1–3.6) | 0 | 0 | 0 | 1 (2) | 0 |
| CA | 4.5 (3.1) | 3.5 (1.4–7.1) | 0.5 (1) | 0.5 (1) | 2.5 (5) | 0 | 0 |
| CG | 2.2 (2.8) | 2.5 (0.8–5.7) | 0 | 0.5 (1) | 1.5 (3) | 0 | 0.5 (1) |
| CL | 2.3 (3.2) | 1.0 (0.1–3.6) | 0 | 1.0 (2) | 0 | 0 | 0 |
| FF | 11.5 (12.0) | 0.5 (0.0–2.8) | 0 | 0 | 0.5 (1) | 0 | 0 |
| GA | 1.4 (1.7) | 0.0 (0.0–1.8) | 0 | 0 | 0 | 0 | 0 |
| GF | 6.4 (5.6) | 1.5 (0.3–4.3) | 0 | 0.5 (1) | 1 (2) | 0 | 0 |
| IW | 1.4 (2.0) | 4.0 (1.7–7.7) | 1.0 (2) | 0 | 2.0 (4) | 0 | 1.0 (2) |
| KN | 2.8 (2.6) | 0.5 (0.0–2.8) | 0 | 0.5 (1) | 0 | 0 | 0 |
| LS | 0.6 (2.3) | 5.0 (2.4–9.0) | 1.0 (2) | 3.0 (6) | 1.0 (2) | 0 | 0 |
| PC | 0.6 (0.8) | 0.0 (0.0–1.8) | 0 | 0 | 0 | 0 | 0 |
| PV | 5.3 (4.8) | 0.5 (0.0–2.8) | 0 | 0.5 (1) | 0 | 0 | 0 |
| CB | 3.5 (2.6) | 2.5 (0.8–5.7) | 0 | 1.0 (2) | 1.0 (2) | 0 | 0.5 (1) |
| MF | 2.6 (3.0) | 1 (0.1–3.6) | 0 | 1.0 (2) | 0 | 0 | 0 |
