# Supplementary material for: Heterogeneity in the abundance and distribution of Ixodes ricinus and Borrelia burgdorferi (sensu lato) in Scotland: implications for risk prediction
Source: Parasit Vectors. 2016 Nov 22;9:595. doi: 10.1186/s13071-016-1875-9 (PMC5120507; doi:10.1186/s13071-016-1875-9)
Supplement: Additional file 2: Table S2. — B. burgdorferi (s.l.) prevalence and genospecies prevalence at each site sampled in, 2007/2008, 2012 and 2013. A full set of genospecies data and sample size to calculate the 95% CI were not available for all sites sampled in 2007/2008. (DOCX 17 kb) [file 13071_2016_1875_MOESM2_ESM.docx]

**Additional file 2. Table S2.** *Borrelia burgdorferi* (*sensu lato*) prevalence and genospecies prevalence at each site sampled in, 2007/8, 2012 and 2013. *Abbreviations*: Bb (*s.l*.) % = overall *B. burgdorferi* (*s.l*.) prevalence, B. val % = *B. valaisiana*, B. gar % = *B. garinii*, B. afz % = *B. afzelii*, B (s.s.) = *B. burgdorferi* (*sensu stricto*), Mixed % = mixed *B. burgdorferi* (*s.l*.). A full set of genospecies data and sample size to calculate the 95% CI were not available for all sites sampled in 2007/8

| **Site name** | **Year** | **Bb (*s.l*.) % (95% C.I.)** | **B.val % (*n*)** | **B.gar % (*n*)** | **B.afz % (*n*)** | **B (*s.s*.) % (*n*)** | **Mixed % (*n*)** |
| --- | --- | --- | --- | --- | --- | --- | --- |
| COM | 2007 | 2 | NA | NA | NA | NA | NA |
| COM | 2012 | 0.5 (0.0–2.8) | 0.5 (1) | 0 | 0 | 0 | 0 |
| COM | 2013 | 5 (2.4–9.0) | 0 | 0 | 4.5 (9) | 0.5 (1) | 0 |
| CR | 2007/8 | 7 | NA | NA | NA | NA | NA |
| CR | 2012 | 1 (0.1–3.6) | 0 | 1.0 (2) | 0 | 0 | 0 |
| CR | 2013 | 7 (3.9–11.5) | 0 | 0.5 (1) | 6.5 (13) | 0 | 0 |
| DR | 2007/8 | 8 | 0.4 | 2.5 | 5 | 0 | 0 |
| DR | 2012 | 12 (7.8–17.3) | 1.0 (2) | 8.0 (16) | 1.0 (2) | 0 | 2 (4) |
| DR | 2013 | 6.5 (3.5–10.9) | 1.0 (2) | 0.5 (1) | 3.5 (7) | 0.5 (1) | 1.0 (2) |
| FZ | 2008 | 14 | 0 | 2 | 11 | 1 | 0 |
| FZ | 2012 | 1 (0.1–3.6) | 0 | 0 | 1 (2) | 0 | 0 |
| FZ | 2013 | 0.5 (0.0-2.8) | 0 | 0 | 0.5 (1) | 0 | 0 |
| TB | 2007/8 | 1 | 0 | 1 | 0 | 0 | 0 |
| TB | 2012 | 0 (0.0–1.8) | 0 | 0 | 0 | 0 | 0 |
| TB | 2013 | 0.5 (0.0–2.8) | 0 | 0.5 (1) | 0 | 0 | 0 |
| WB | 2007/8 | 2 | NA | NA | NA | NA | NA |
| WB | 2012 | 4 (1.7–7.7) | 0 | 0.5 (1) | 3.5 (7) | 0 | 0 |
| WB | 2013 | 3.5 (1.4–7.1) | 0 | 0 | 3.5 (7) | 0 | 0 |

NA, Not available
