# Supplementary material for: Heterogeneity in the abundance and distribution of Ixodes ricinus and Borrelia burgdorferi (sensu lato) in Scotland: implications for risk prediction
Source: Parasit Vectors. 2016 Nov 22;9:595. doi: 10.1186/s13071-016-1875-9 (PMC5120507; doi:10.1186/s13071-016-1875-9)
Supplement: Additional file 3: Table S3. — Results for the final generalised linear mixed model to explain variation in the prevalence of infected nymphs collected at six sites sampled in 2012 and 2013. (DOCX 14 kb) [file 13071_2016_1875_MOESM3_ESM.docx]

**Additional file 3: Table S3.** Results for the final generalised linear mixed model to explain variation in the prevalence of infected nymphs collected at six sites sampled in 2012 and 2013.

| **Model description** | **Fixed Effects** | **Mean (estimated)** | **Standard Error** | ***P*-value** | **Delta AICc** |
| --- | --- | --- | --- | --- | --- |
| *B. burgdorferi* (*s.l*.) | Intercept | -3.9 | 0.5 | < 2.0 × 10^-16^ | – |
| *B. afzelii* | Intercept | -5.3 | 0.7 | 1.4 × 10^-15^ | – |
|  | Year (2013) | 1.5 | 0.8 | 0.061 | 1.6 |
| *B. garinii* | Intercept | -6.2 | 0.8 | 1.6 × 10^-13^ | – |
