# Supplementary material for: Heterogeneity in the abundance and distribution of Ixodes ricinus and Borrelia burgdorferi (sensu lato) in Scotland: implications for risk prediction
Source: Parasit Vectors. 2016 Nov 22;9:595. doi: 10.1186/s13071-016-1875-9 (PMC5120507; doi:10.1186/s13071-016-1875-9)
Supplement: Additional file 4: Table S4. — Results from the final selected generalised linear mixed model to explain variation in questing Ixodes ricinus nymph abundance. Delta AICc indicates the change in AICc after removing each variable from the best-fit model. (DOCX 14 kb) [file 13071_2016_1875_MOESM4_ESM.docx]

**Additional file 4. Table S4.** Results from the final selected generalised linear mixed model to explain variation in questing *Ixodes ricinus* nymph abundance. Delta AICc indicates the change in AICc after removing each variable from the best-fit model

| **Fixed effects** | **Mean (estimated)** | **Standard Error** | ***P*-value** | **delta AICc** |
| --- | --- | --- | --- | --- |
| (Intercept) | -1.74 | 0.92 | 0.058 | – |
| Ground vegetation (grass)* | 0.5 | 0.21 | 0.017 | 2.7 |
| Growing degree-days** | 0.18 | 0.08 | 0.021 | 2.5 |

*Grass relative to other ground vegetation types: ericaceous, bracken and moss

**UK Met Office Long Term Average climate data [35]
